# Comparison of letrozole with gonadotropin-releasing hormone agonist in frozen embryo transfer after recurrent implantation failure: An RCT

**DOI:** 10.18502/ijrm.v18i2.6417

**Published:** 2020-02-27

**Authors:** Nayere Khadem Ghaebi, Malihe Mahmoudiniya, Mona Najaf Najafi, Elnaz Zohdi, Matin Attaran

**Affiliations:** ^1^Department of Obstetrics, Faculty of Medicine, Mashhad University of Medical Sciences, Mashhad, Iran.; ^2^Imam Reza Clinical Research Units, Faculty of Medicine, Mashhad University of Medical Sciences, Mashhad, Iran.

**Keywords:** Letrozole, Fertilization in vitro, Pregnancy outcome.

## Abstract

**Background:**

The use of frozen embryo transfer (FET) is increasing worldwide in the treatment of infertility by in vitro fertilization. Different methods of endometrial preparation for FET have been suggested.

**Objective:**

The aim of this study was to compare the pregnancy outcomes after treatment with letrozole and those after treatment with the combination of gonadotropin-releasing hormone (GnRH) agonist and estradiol in FET.

**Materials and Methods:**

This randomized controlled trial study was conducted on 142 infertile women with a history of previous FET failure. Participants were randomly assigned to two groups (n = 71 each). The GnRH group received 500 µg of buserelin plus 4mg estradiol (which increased to 8 mg if endometrial thickness was less than 5 mm), and the letrozole group received 5 mg of letrozole plus 75 IU of recombinant human follicle-stimulating hormone). At least two high-quality embryos were transferred to each subject in both groups. The outcome measures were clinical pregnancy rate and fetal heart rate detection.

**Results:**

Subjects in the study groups had similar demographic characteristics and baseline clinical condition. Mean endometrial thickness in the letrozole and GnRH agonist groups were 8.90 ± 0.88 mm and 8.99 ± 0.85 mm, respectively (p = 0.57). The number of positive results of the beta human chorionic gonadotropin test and detection of fetal heartbeat were not significantly different between the groups (p > 0.05).

**Conclusion:**

The administration of letrozole and GnRH may produce similar pregnancy outcomes in FET.

## 1. Introduction

Frozen embryo transfer (FET) has become a successful technique for in vitro fertilization (IVF). An important factor in the success in IVF is the proper synchronization between the fetus and endometrial growth (1-5). Previous studies have shown that endometrial preparation before FET was linked to better outcomes than was FET performed during the normal cycle (6, 7). The reason for the higher failure in FET in normal cycles might be the difficulty in identifying the optimal time for FET because of the irregularity in menstruation in some women and the difficulty in the assessment of the endometrial preparation (8). Therefore, different methods are used to prepare the endometrium for FET, including artificial-cycle FET (9-11).

A common method for artificial endometrium preparation is the use of estrogen and progesterone hormones with or without gonadotropin-releasing hormone (GnRH) agonist (9-11). This method (GnRH plus estrogen and progesterone) was shown to be effective especially in cases of previous IVF failures and may decrease the number of embryo transfer failures (9-11). An alternative to the use of GnRH agonists for endometrial induction is the use of letrozole (12). Letrozole is a third-generation aromatase inhibitor that does not have negative endometrial effects and preserves the normal response of the central feedback system for follicular growth and ovulation (12). In comparisons of the effect of letrozole and GnRH agonists on the FET outcome in infertile women, the findings have been controversial (7, 13, 14).

The aim of this study was to compare letrozole with combination therapy with GnRH agonist and estradiol valerate for endometrial induction in FET among infertile women with a history of FET failure.

## 2. Materials and Methods

**Figure 1:**
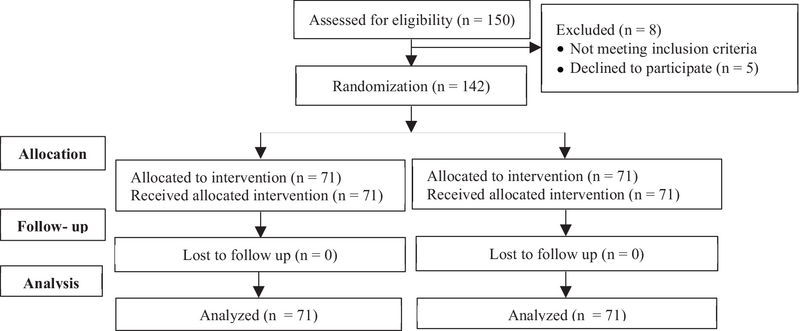
Patient assignment in study.

This randomized controlled trial was conducted in Milad Infertility Center, Mashhad, Iran, with 150 women who were referred for FET; 142 of them entered this study in January 2019 (Figure 1).

The inclusion criteria were willingness to participate in the study by giving written informed consent, age between 18 and 35 years, and a history of at least two cycles of FET failure. Women were excluded if severe infertility had been diagnosed in a spouse, if severe endometriosis (grade 3 or 4) were present, if a fibroma larger than 4 cm were present, or if the women were candidates for testicular sperm extraction for failure to otherwise produce a live embryo.

Demographic data were collected from patients' medical records. Subjects were randomly divided into two groups based on simple random sampling method, where the first subject was randomly assigned to GnRH group and the consecutive subjects were assigned to the other group until the sample size was reached for each group. Subjects in the GnRH agonist group received 500 µg of buserelin daily subcutaneously from day 21 of the menstrual cycle. Estradiol was initiated at the time of cycle in the GnRH agonist group at a dose of 4 mg and increased to 8 mg if the endometrial thickness was less than 5 mm or the follicular diameter was less than 10 mm. Subjects underwent an ultrasound scan on day 3 of the next menstrual cycle. The first ovarian monitoring ultrasound examination was performed on day 12 of the menstruation cycle, at which point 600 mg of vaginal progesterone (Utrogestan; Besins Manufacturing Belgium, Brussels, Belgium) was administered. Subjects in the letrozole group received 2.5 mg of letrozole (Aburaihan Co., Tehran, Iran) daily, beginning on day 3 of the menstrual period, for 5 days. Subjects were then administered 75 IU of recombinant human follicle-stimulating hormone (CinnaGen Co., Tehran, Iran) subcutaneously starting on day 9 until the follicular size reached 18 mm. Then 10,000 IU of human chorionic gonadotropin (β-CG; Karma Pharmacology, Germany) was administered intramuscularly to induce ovulation; 2 days later, subjects began receiving progesterone, administered for 5 days at the dose of 100 mg/day to enhance formation of a blastocyst.

The quality of embryos was checked daily. Each 5-day old embryo was incubated in Blastocyst Medium (Origio, Måløv, Denmark). Embryos were defined as being of grade A quality if four blastomeres on day 2 or if seven or eight blastomeres were observed on day 3 with equal size and no more than 20% fragmentation, according to Gardner *et al*. criteria (15). Under ultrasound guidance, the Cook catheter (Cook Medical, Eight Mile Plains, Queensland, Australia) was used to transfer one or two embryos of the best quality. The most developed embryo was transferred in case no blastocyst existed on day 5. Serum β-hCG level was assessed 14 days after embryo transfer. Clinical pregnancy was defined as the observation of a gestational sac 6 weeks after embryo transfer. To calculate the implantation rate, the number of gestational sacs in all subjects was divided by the total number of transferred embryos. Continued pregnancy was defined as the presence of pregnancy until the 12th week of gestation.

### Ethical consideration

The study protocol was approved by the Ethical Committee of the Mashhad University of Medical Sciences, Mashhad, Iran (Reg. no. IR.MUMS.MEDICAL.REC.1397.412). Subjects were approached by the researchers and received information regarding the aim and procedure of the study. Subjects who were willing to participate in the study were asked to sign an informed consent form.

### Statistical analysis

Data were analyzed with SPSS software version 22 (IBM, Inc., Chicago, IL, USA). Continuous variables were checked for normality with the Shapiro-Wilk test. Normally distributed variables were calculated as means and standard deviations, whereas nonnormally distributed variables were calculated as medians with interquartile ranges. Categorical variables were calculated as frequencies and percentages. The distribution patterns of categorical variables of the groups were compared with the chi-square or Fisher's exact test; while continuous variables were compared with the independent Student's t test or the Mann-Whitney U test. The level of significance was defined as a p value smaller than 0.05.

## 3. Results

A total of 142 subjects participated in the study. The demographic characteristics of the subjects are listed in Table I. There was no significant difference in the distribution pattern of demographic characteristics between study groups. Of the subjects, 5 (3.5%) reported a history of using medications other than infertility medications, including levothyroxine in 4 (2.8%) and insulin in 1 (0.7%).

**Figure 2:**
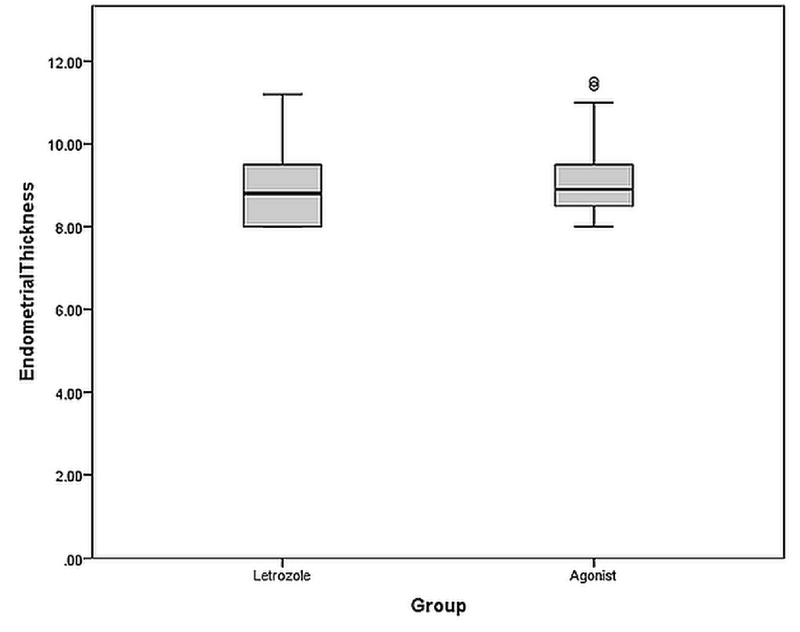
Endometrial thickness in the study groups. The plot indicates one subject had endometrial thickness more than 2 standard deviation higher than the mean (marked as θ).

Mean endometrial thickness in the letrozole and GnRH agonist groups were 8.90 ± 0.88 mm and 8.99 ± 0.85 mm respectively. There was no significant difference in terms of endometrial thickens between study groups (p = 0.57) (Figure 2).

The prevalence of positive β-hCG test and detectable fetal heart rate were not significantly different between groups (Table II).

**Table 1:** Demographic characteristics of subjects by study group


**Characteristic**			**Total (n = 142)**	**Letrozole group (n = 71)**	**GnRH agonist group (n = 71)**	**p-value**
**Infertility type**						
	**Primary**		122 (85.9%)	62 (87.3%)	60 (84.5%)	
	**Secondary**		20 (14.1%)	9 (12.7%)	11 (15.5%)	0.63†
**Cause of infertility**						
	**Male (oligospermia)**		44 (31.0%)	24 (33.8%)	20 (28.2%)	
	**Female cause**		40 (28.2%)	18 (25.4%)	22 (31.0%)	
	**Unknown**		58 (40.8%)	29 (40.8%)	29 (40.8%)	0.68†
**Gravida**						
	**1**		14 (9.9%)	7 (100.0%)	7 (77.8%)		
	**2**		2 (1.4%)	0 (0.0%)	2 (22.2%)	0.47†
**History of abortion**			10 (7.0%)	3 (4.2%)	7 (9.9%)		0.19‡
**Age**			29.74 ± 4.38	29.86 ± 4.46	29.62 ± 4.33	0.75††
**BMI**			24.37 ± 2.90	24.37 ± 2.91	24.37 ± 2.91	1.00††
**Infertility duration (years)**			4.00 (3.25)	4.00 (4.00)	4.00 (3.00)	0.37‡
**Number of abortions**			1.1 ± 0.33	1.00 ± 0.00	1.22 ± 0.44	0.54††
**IVF trials**			1.12 ± 0.33	1.07 ± 0.26	1.17 ± 0.38	0.07††
**Number of unsuccessful FETs**			2.05 ± 0.81	2.04 ± 0.82	2.06 ± 0.81	0.92††
**Number of unsuccessful fresh embryo transfers**			1.05 ± 0.32	1.06 ± 0.23	1.04 ± 0.39	0.79††
† Data presented as Frequency (%). Chi-square test †† Data presented as Mean ± SD. Independent t test ‡Data presented as medians and interquartile ranges. Mann-Whitney test BMI = Body mass index; FET = Frozen embryo transfer; IVF = In vitro fertilization; GnRH = Gonadotropin-releasing hormone. †† Median and interquartile range (IQR) were used for presentation and the Mann-Whitney test was used for the comparison						

**Table 2:** Comparison of study findings between groups


**Tested parameter**		**Letrozole group (n = 71)**	**GnRH agonist group (n = 71)**	**p-value**
**β-hCG test result**				
	**Positive**	6 (8.4%)	5 (7.0%)	
	**Negative**	65 (91.6%)	66 (93.0%)	0.73
**Fetal heart rate**				
	**Present**	4 (66.6%)	3 (60.0%)	
	**Absent**	2 (33.4%)	2 (40.0%)	0.82
Data presented as Frequency (%). Chi-square test β-hCG = β-human chorionic gonadotropin; GnRH = Gonadotropin-releasing hormone. Fisher's exact test was used for the comparison				

## 4. Discussion

The findings of this study revealed that the mean endometrial thicknesses did not significantly differ between the two groups; it was slightly higher in the GnRH agonist group (0.09 mm on average). Previous studies have yielded similar findings, which indicates that both preparation methods may increase endometrial thickness to a similar extent (7, 13). In a previous study, Huang and colleagues performed endometrial preparation by administering letrozole, followed by either GnRH agonist or β-hCG (13). They found no significant difference in endometrial thickness between patients receiving letrozole and those receiving GnRH agonist, although the endometrial thicknesses that they reported were higher than that observed in our study (10.77 mm in patients receiving letrozole and GnRH, 10.61 mm in those receiving letrozole and β-hCG) (13). The difference in the endometrial thickness between the two studies might have resulted from differences in the preparation method, inasmuch as both groups in Huang and colleagues' study received letrozole, whereas in our study, letrozole was administrated to only one group. Aleyasin and coworkers observed no significant difference in endometrial thickness between patients receiving letrozole and those receiving GnRH group, and the mean endometrial thicknesses in their patients (8.08 mm in those receiving letrozole and in those receiving GnRH) were slightly lower than those observed in our study (7). It was previously shown that endometrial thickness and endometrial pattern were not associated with pregnancy outcome (16).

The rate of pregnancy achievement in this study was 8.4% in the letrozole group and 7.0% in the GnRH group. This pregnancy rate was lower than the 18.5% to 53.1% reported in previous studies (7, 10, 13, 17). The difference in the pregnancy rates between our study and the previous studies might have resulted from differences in the methodologies, study populations, duration of infertility, and comorbidities among study subjects. Furthermore, our study revealed no significant difference between the groups in rate of pregnancy achievement and FHR. This finding was in line with the findings of previous studies (7, 13, 18, 19). In a previous study, the pregnancy rate was significantly higher among patients receiving letrozole, but the rates of clinical pregnancy and pregnancy continuation did not differ significantly between the methods (20). Letrozole causes downregulation of estrogen production by inhibition of cytochrome P450, which in part results in increased secretion of follicle-stimulating hormone from the pituitary gland and in folliculogenesis (21). Letrozole also does not affect the central negative feedback of estrogen (21).

Our study revealed no significant difference in the rates of pregnancy and continuation of pregnancy between groups; however, because of the low pregnancy rate, the findings of this study may not apply to whole populations. Further studies with a larger sample size are needed in order to identify the pregnancy rate in FET cases in Iran.

## 5. Conclusion

Letrozole administration for endometrial preparation for FET might be as effective as GnRH administration.

##  Conflicts of Interest 

None
